# The Mayo Clinic Florida Microdosimetric Kinetic Model of Clonogenic Survival: Application to Various Repair-Competent Rodent and Human Cell Lines

**DOI:** 10.3390/ijms232012491

**Published:** 2022-10-18

**Authors:** Alessio Parisi, Chris J. Beltran, Keith M. Furutani

**Affiliations:** Department of Radiation Oncology, Mayo Clinic, Jacksonville, FL 32224, USA

**Keywords:** clonogenic survival, microdosimetry, particle therapy, MCF MKM, relative biological effectiveness, PHITS

## Abstract

The relative biological effectiveness (RBE) calculations used during the planning of ion therapy treatments are generally based on the microdosimetric kinetic model (MKM) and the local effect model (LEM). The Mayo Clinic Florida MKM (MCF MKM) was recently developed to overcome the limitations of previous MKMs in reproducing the biological data and to eliminate the need for ion-exposed in vitro data as input for the model calculations. Since we are considering to implement the MCF MKM in clinic, this article presents (a) an extensive benchmark of the MCF MKM predictions against corresponding in vitro clonogenic survival data for 4 rodent and 10 cell lines exposed to ions from ^1^H to ^238^U, and (b) a systematic comparison with published results of the latest version of the LEM (LEM IV). Additionally, we introduce a novel approach to derive an approximate value of the MCF MKM model parameters by knowing only the animal species and the mean number of chromosomes. The overall good agreement between MCF MKM predictions and in vitro data suggests the MCF MKM can be reliably used for the RBE calculations. In most cases, a reasonable agreement was found between the MCF MKM and the LEM IV.

## 1. Introduction

Cancer radiotherapy treatments with ions are optimized to account for their different relative biological effectiveness (RBE) with respect to conventional X-rays [1]. The modelled endpoint considered for treatment planning is the cellular clonogenic survival [2] due to its relevance for clinical tumor control calculations [3]. Among the biophysical models developed during the years [4,5,6,7,8,9,10,11,12,13,14,15] , mainly two approaches are currently in clinical use to calculate these RBE variations: the first version of the local effect model (LEM I [6]) based on amorphus track structure calculations and the modified microdosimetric kinetic model (modified MKM [9]) based on microdosimetry. It must be mentioned that phenomenological models such as the mixed beam model (MBM [7]) are in use in some carbon therapy facilities [16]. In the MBM [7], ion-specific empirical correlations between the average linear energy transfer (LET) and the linear and quadratic terms (α and *ß*) of the linear quadratic model (LQM [17,18]) are established by fitting the results of in vitro experiments and used for the biophysical calculations [7,19]. 

While amorphus track structure models such as the LEM [6] rely on the computation of the radial dose distribution around the ion track, microdosimetric models (for example the modified MKM [9]) are based on quantities such as the lineal energy which be simulated and experimentally measured with dedicated radiation detectors [20]. Consequently, treatment planning calculations with microdosimetric models possess the advantageous possibility of an experimental validation by means of independent physical measurements.

Despite some limitations of the LEM I [6] were reported [21,22,23], this is the only LEM version currently implemented in clinical practice. In order to improve the agreement between the model calculations and the biological data, subsequent corrections for the clustering of deoxyribonucleic acid (DNA) single strand breaks at the nanometer level and an improved description of the radial dose distribution were introduced in the LEM II [24] and further modified in the LEM III [25]. The fourth version of the LEM [26] is a major update of the LEM. While in the previous LEM versions [6,24,25] the radial dose distribution is used as the operational quantity for the biophysical modeling process, the LEM IV [26] includes an intermediate step in the calculation of the radiation induced damage. Here, the radial dose distribution is used to evaluate the local distribution of DNA double strand breaks (DSBs) in the cell nucleus. Afterwards, a measure of the DNA DSB clustering within subnuclear volumes (i.e., the cluster index [26]) is derived and then used to compute the cell survival. The various LEM parameters [27] were determined during the years by fitting a subset of in vitro data. Since it is generally assumed that these model parameters can be unchangingly applied to any cell line, the only input for the LEM calculations is the dose–response after photon irradiation [28]. 

The original version of the MKM [5], developed from the theory of dual radiation action [4], does not account for the experimentally observed decrease in the relative biological effectiveness (RBE) at high linear energy transfer (LET) due to overkill effect [29]. In the modified MKM [9], the overkill effect is included in the model formalism by means of a phenomenological quantity (the saturation-corrected dose-mean lineal energy [30]) evaluated in subnuclear structures of the cell nucleus named domains. However, it was shown that the modified MKM possesses limitations in reproducing the in vitro data high LET and low surviving fractions [9,11,31]. These deviations are due to a suboptimal implementation of the overkill effects in the modeling of the linear term (α) of the LQM [31] and to the model assumption of a radiation independent quadratic term (*ß*) of the LQM [9,11,31]. 

In view of the upcoming integrated proton-carbon ion therapy center at Mayo Clinic Florida (MCF, Jacksonville, Florida, United States of America [32]), a novel microdosimetric model named MCF MKM was recently developed [33]. Differently from previous versions of the MKM [5,8,9], the MCF MKM introduces novel expressions to calculate the exposure-specific values of α and *ß* by using whole spectral information. Additionally, the mean radius of the radiation-sensitive subnuclear domains is a priori calculated using a new methodology based on the population-mean amount of DNA content in the cell nucleus [33]. This is an important difference between the MCF MKM and the previous MKMs. In the latter case, the cell-specific parameters are generally assessed a posteriori by fitting a subset of the in vitro data in case of ion exposures, thus partially limiting the predictive power of these models. On the other hand, without using any in vitro ion-exposure data as input to the MCF MKM nor performing a posteriori tuning of the model parameters (mean radius of the cell nucleus *R*_n_, mean radius subnuclear domains *r*_d_), the MCF MKM was successful in predicting average and experiment-specific RBE trends for the most commonly used mammalian cell line (Chinese hamster lung fibroblasts, V79 cell line) and the clinically relevant human salivary gland tumor cells (HSG cell line) for ions from ^1^H to ^132^Xe. A sensitivity test proved that the a priori determined MCF MKM parameters for these two cell lines were in good agreement with the ones obtained by fitting the in vitro data [33]. 

Considering a possible clinical implementation of the MCF MKM, it is of primordial importance to (a) validate the model predictions against a large dataset of in vitro clonogenic survival data for various cell lines and (b) compare the MCF MKM results with the results of the most recent version of clinically relevant models such as the LEM. Therefore, in this article we investigate the accuracy of the MCF MKM in systematically predicting the LQM terms and the RBE values for 4 rodent (Chinese hamster, mouse, rat) and 10 human repair-competent cell lines in case of exposures to ions from ^1^H to ^238^U. Since morphologic measurements of the cell nucleus (*R*_n_) are not always available in the literature, this work also presents a phenomenological approach to derive an approximate value of both model parameters (mean radius of the cell nucleus *R*_n_, mean radius subnuclear domains *r*_d_) by knowing the animal species and, for aneuploid cancer cells, the mean number of chromosomes. When possible, the MCF MKM prediction were compared with published results of the LEM IV [28]. 

## 2. Results and Discussion

In the following paragraphs, the linear and quadratic terms of the LQM (α and *ß*) and the RBE for a surviving fraction of 10% (RBE_10%_) predicted by the MCF MKM are compared with the experimental in vitro data from PIDE 3.2 [34] and published calculations [28] with the latest version of the local effect model (LEM IV [26]). Since the LEM IV calculations are experiment-specific, the LET-dependence of the calculated RBE values could show a non-monotonic behavior between subsequent points. The dashed lines for the LEM IV data series are meant as a guide to the eye only. The results are plotted as a function of the unrestricted LET in water for the different ions and cell lines included in this investigation. In vitro and in silico data for different isotopes of the same element (i.e., ^1^H and ^2^H ions) were pooled together since negligible differences were observed when the results are plotted as a function of the LET [35,36].

Though the MCF MKM can be used to compute the RBE also for other surviving fractions, the plots are limited to the RBE_10%_ for better clarity in the comparison between the three data series (MCF MKM, in vitro, LEM IV). Nonetheless, examples of RBE calculations for other surviving fractions (50% and 1%) are given in Figures S1–S14 in the Supplementary Materials. 

The PIDE in vitro database does not provide uncertainty intervals for the clonogenic survival data. This is due to the fact that most of the published biological studies do not include an uncertainty analysis and, in some cases, not even an indication of the statistical dispersion of the results. The RBE results for the clonogenic survival essay are affected by several sources of uncertainty, such as: the different operational protocols between different groups (as an example, the time between irradiation and plating is known to play a significant effect on the clonogenic survival [37]), cell line aging [29], cell misidentification or cross-contamination [38,39], the process of colony counting (15–30% [40]), the choice of the reference radiation (details on the photon energy spectrum, very important for filtered X-rays, are generally not listed [36]), the lack of standard procedures to calibrate biological irradiators [41,42], and the processing of fitting the in vitro data to obtain the survival curve (for the LQM this fitting process is significantly affected by the anti-correlation between α and *ß* [34]). All of above, together with a widespread incomplete report of physical parameters [43], is to be held responsible for the large scatter in the in vitro RBE data obtained in comparable conditions (i.e., the same cell line exposed to a similar radiation quality [34,36]) and thus the reproducibility crisis in radiobiological studies [44]. Consequently, since an accurate retrospective uncertainty analysis dealing with both stochastic and systematic error is currently not possible, the in vitro data are plotted without error bars. 

### 2.1. Rodent Cell Lines

**Figure 1:**
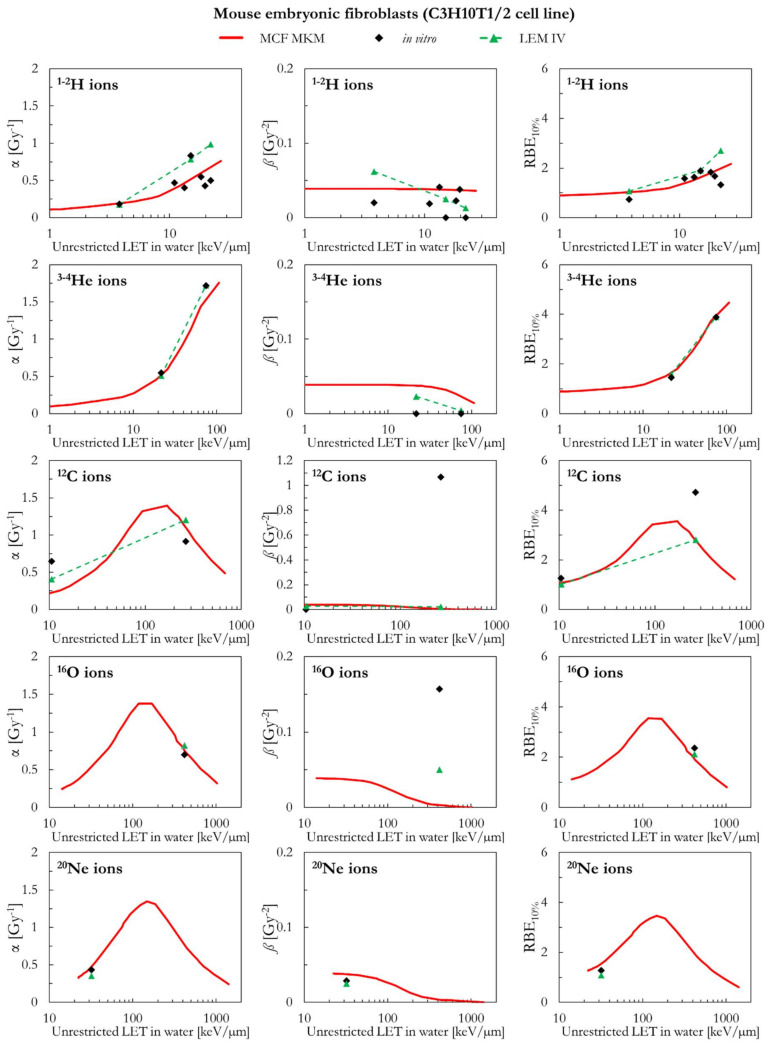
α, *ß*, and RBE_10%_ for the C3H10T1/2 cell line in case of ^1-2^H, ^3-4^He, ^12^C, ^16^O, and ^20^Ne ions: MCF MKM predictions compared with published in vitro data from PIDE 3.2 [34] and published LEM IV results [28].

**Figure 2:**
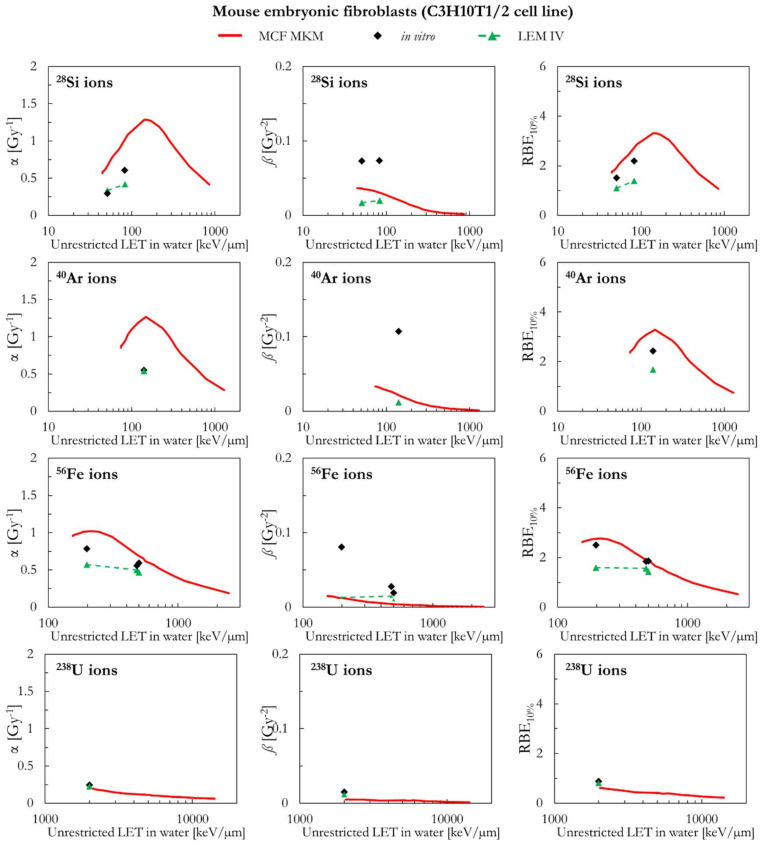
α, *ß*, and RBE_10%_ for the C3H10T1/2 cell line in case of ^28^Si, ^40^Ar, ^56^Fe and ^238^U ions: MCF MKM predictions compared with published in vitro data from PIDE 3.2 [34] and published LEM IV results [28].

The results for mouse embryonic fibroblasts (C3H10T1/2 cell line) are shown in Figure 1 and Figure 2. Except for ^1^H ions where the LEM IV seems to predict higher α and RBE_10%_ values, the results of the calculations with the two models are in good agreement between each other and with the in vitro data of Figure 1 (ions from ^1^H to ^20^Ne). It is worth remembering that the prediction of large *ß* values (i.e., the ones for the in vitro data around 4–500 keV/µm for ^12^C and ^16^O ions) is beyond the capabilities of both models. Furthermore, it should be noted that, here and in the following, the *ß* values predicted by the LEM show a sharper decrease with the increase of the LET with respect to the *ß* values calculated with the MCF MKM. In case of ^28^Si and heavier ions (Figure 2), the agreement between the data series is less striking. For ^28^Si and ^40^Ar ions, the α calculated with the LEM IV appear to be closer to the in vitro data than the MCF MKM results. Nonetheless, the MCF MKM seems to better reproduce the RBE_10%_ values in case for ^28^Si and ^56^Fe ions. In case of ^238^U ions, both models reproduce reasonably well the in vitro data. 

**Figure 3:**
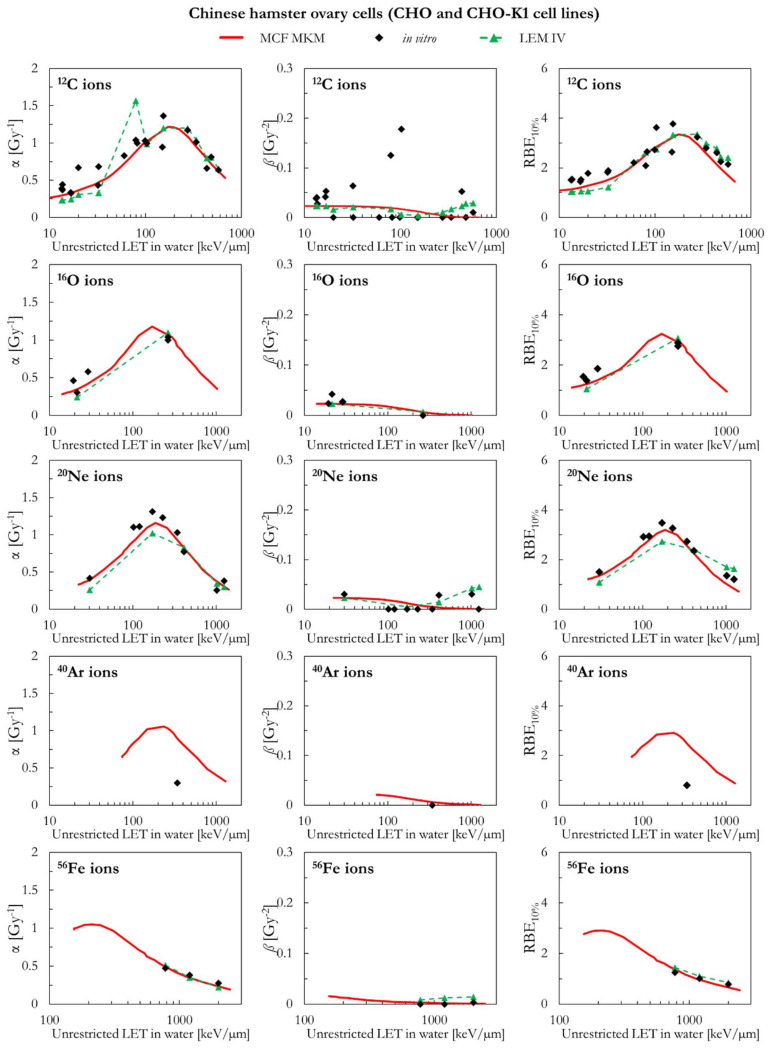
α, *ß*, and RBE_10%_ for the CHO and the CHO-K1 cell lines in case of ^12^C, ^16^O, ^20^Ne, ^40^Ar, and ^56^Fe ions: MCF MKM predictions compared with published in vitro data from PIDE 3.2 [34] and published LEM IV results [28].

The MCF MKM and the LEM IV results appear to be in good agreement with the in vitro data for chinese hamster ovary cells (CHO and CHO-K1 cell lines) over the whole investigate ion-LET range (Figure 3). The only exception is the one data point for ^40^Ar ions. In this case, the MCF MKM predictions appear to be higher than the in vitro data. No LEM IV calculations for the CHO cell line were available for ^40^Ar ions. The somehow counterintuitive increase of the LEM-calculated *ß* term at very high LET is due to the current implementation of the DNA damage enhancement factor [28].

**Figure 4:**
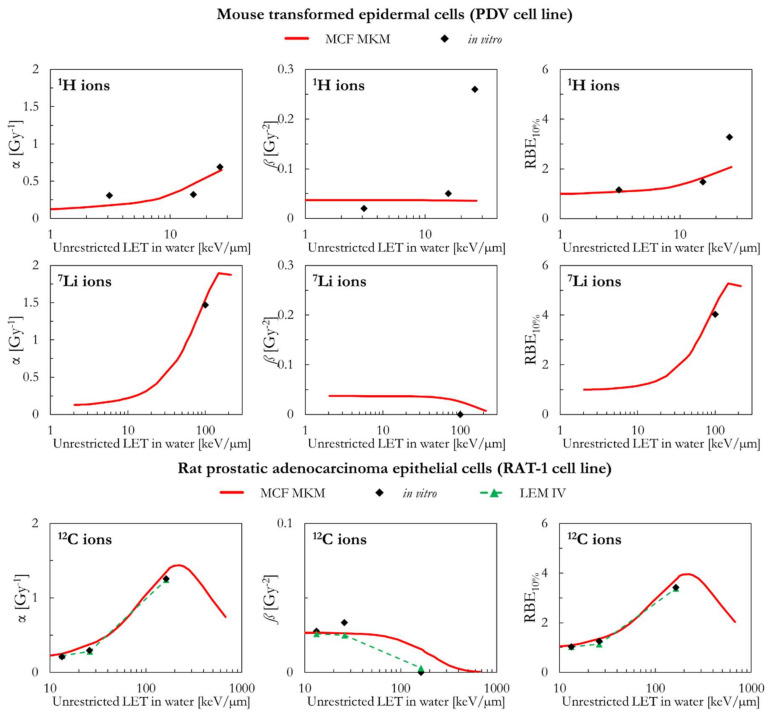
α, *ß*, and RBE_10%_ for the PDV and the RAT-1 cell lines in case of ^1^H (PDV cell line), ^7^Li (PDV cell line), and ^12^C (RAT-1 cell line) ions: MCF MKM predictions compared with published in vitro data from PIDE 3.2 [34] and published LEM IV results [28].

The MCF MKM predictions for transformed mouse epidermal cells (PDV cell line) are plotted in Figure 4 for ^1^H and ^7^Li ions. Except for the larger in vitro *ß* data point for 26 keV/µm protons (*ß* = 0.26 Gy^−2^, roughly 7 times higher than *ß*_ref_ = 0.037 Gy^−2^), the model calculations (α, *ß* and RBE) are in good agreement with the in vitro data for both ^1^H and ^7^Li ions. Due to the approach used in Equation (4), the MCF MKM always predicts a monotonic decrease of *ß* with the increase of the particle LET (i.e., *ß*/*ß*_ref_ ≤ 1). 

The α values predicted by MCF MKM and the LEM IV for carbon-irradiated rat prostatic adenocarcinoma epithelial cells (RAT-1 cell line, Figure 4) are very similar and agree remarkably well with the corresponding in vitro data. Though the MCF MKM appears to overestimate the *ß* value around 160 keV/µm, this has a little effect on the calculated RBE_10%_ since the α term is significantly larger than *ß*. As for α, the RBE_10%_ calculated with both models are similar and in good agreement with the experimental data.

### 2.2. Human Cell Lines

**Figure 5:**
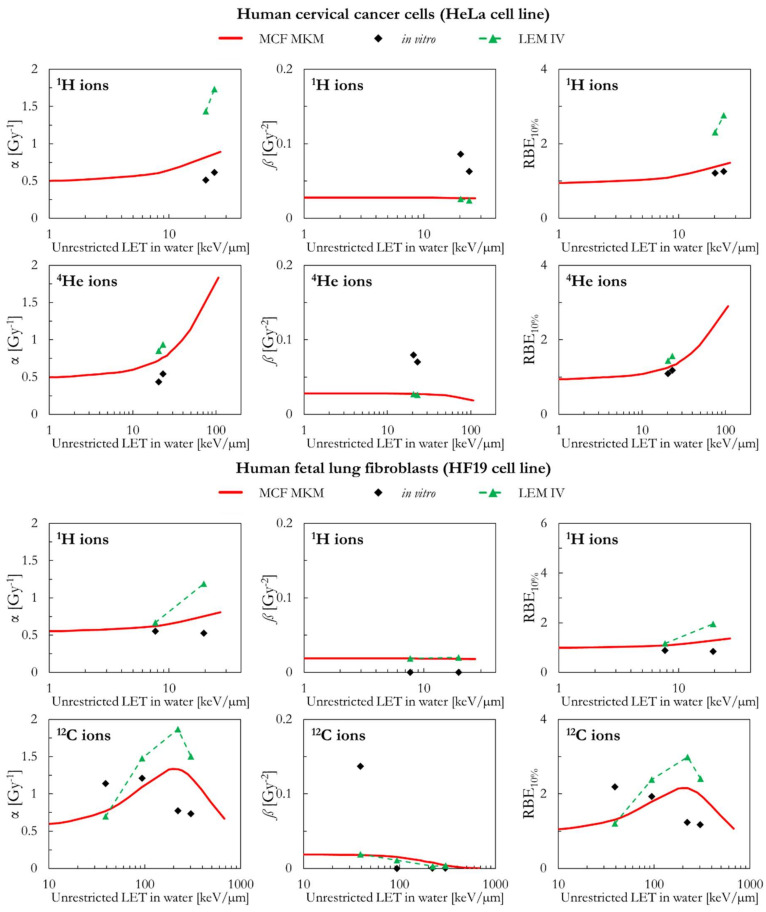
α, *ß*, and RBE_10%_ for the HeLa and the HF19 cell lines in case of ^1^H (HeLa and HF19 cell lines), ^4^He (HeLa cell line), and ^12^C (HF19 cell line) ions: MCF MKM predictions compared with published in vitro data from PIDE 3.2 [34] and published LEM IV results [28].

The results of human cervical cancer cells (HeLa cell line) are plotted in Figure 5 as a function of the LET for ^1^H and ^4^He ions. Both models (MCF MKM and LEM IV) overestimate the in vitro α values for ^1^H ions. The overestimation is significantly larger for the LEM IV. By contrast, the proton *ß* values calculated by the two models agree well between each other but underestimate the in vitro *ß* data. This concomitant overestimation of α and underestimation of *ß* might be an artifact due to the anti-correlation between the two LQM terms during the fit of the in vitro data [34,45]. Nonetheless, the RBE_10%_ values predicted by the MCF MKM are in good agreement with the corresponding in vitro results for ^1^H ions. By contrast, the LEM IV seems to overestimate the HeLa proton RBE_10%_. Though the results of the three data series (MCF MKM, LEM IV, in vitro) are in closer agreement, the discussion of the HeLa results for ^4^He ions follows the one for ^1^H ions and it is not repeated. 

As for the HeLa cell line, the α and RBE_10%_ calculated by the two models (MCF MKM and LEM IV) in case of proton-irradiated human fetal lung fibroblasts (HF19 cell line) are higher than the corresponding in vitro data (Figure 5). The overestimation is larger for the LEM IV. The in silico *ß* values for ^1^H ions are in good agreement between MCF MKM and LEM IV, but significantly higher than the in vitro ones. For ^12^C ions, the unclear trend of the in vitro data prevents any meaningful discussion on the biophysical calculations. 

**Figure 6:**
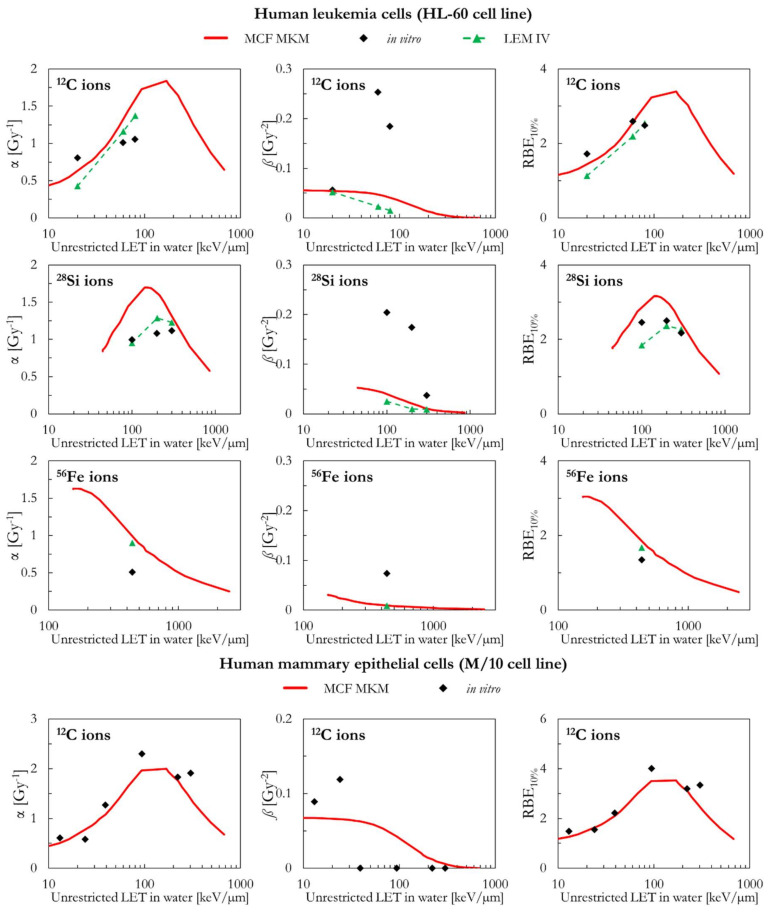
α, *ß*, and RBE_10%_ for the HL-60 and the M/10 cell lines in case of ^12^C (HL-60 and M/10 cell lines), ^28^Si (HL-60 cell line), and ^56^Fe (HL-60 cell lines) ions: MCF MKM predictions compared with published in vitro data from PIDE 3.2 [34] and published LEM IV results [28].

Figure 6 includes a comparison between the in silico and in vitro data for human leukemia cells (HL-60 cell line) for ^12^C, ^28^Si, and ^56^Fe ions. In all cases, the *ß* values by the MCF MKM and the LEM IV are significantly smaller than the in vitro data. Therefore, due the previously discussed anti-correlation, it is expected that the modelled α values are overestimated by the models. Except for a ^12^C data point at ~20 keV/µm, this seems the case for the MCF MKM in case of ^12^C and ^56^Fe ions. Nonetheless, the RBE_10%_ is reasonably well described by both models for ^12^C and ^56^Fe ions. For ^28^Si ions, the MCF MKM appear to systematically overestimates the α and the RBE_10%_ values. By contrast, the LEM IV well reproduces the α values for ^28^Si ions. Furthermore, the LEM IV underestimates the RBE_10%_ for the 100 keV/µm ^28^Si ions, but well describes the in vitro data at higher LET (2–300 keV/µm ^28^Si ions). 

The MCF MKM results for human mammary epithelial cells (M/10 cell line) are shown in Figure 6 together with the corresponding in vitro data for ^12^C ions. No LEM IV data were available. A good agreement between the calculated and the measured cell response is present over the whole LET range. 

**Figure 7:**
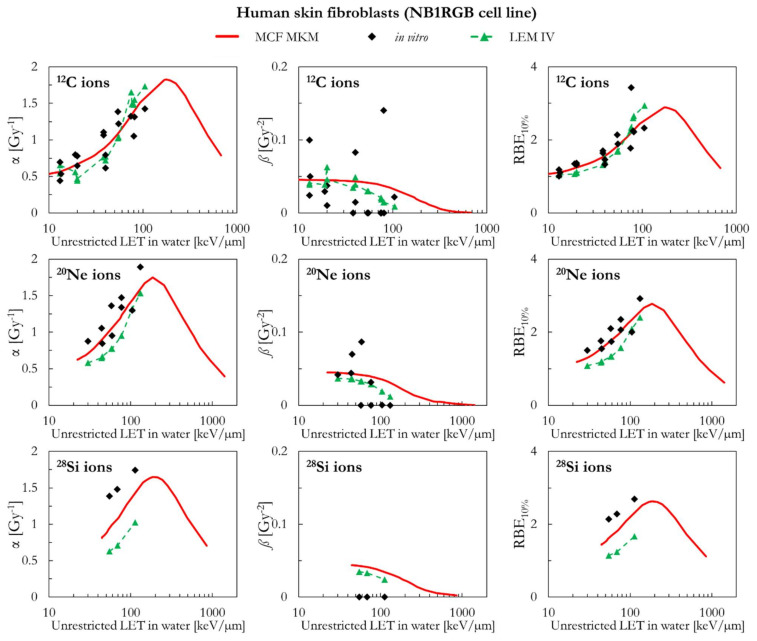
α, *ß*, and RBE_10%_ for the NB1RGB cell line in case of ^12^C, ^20^Ne, and ^28^Si ions: MCF MKM predictions compared with published in vitro data from PIDE 3.2 [34] and published LEM IV results [28].

Figure 7 compares the in silico and in vitro results for human skin fibroblasts (NB1RGB cell line) in case of exposures to ^12^C, ^20^Ne, and ^28^Si ions. For ^12^C ions, the α and RBE_10%_ values calculated with the MCF MKM and the LEM IV are reasonably similar and in good agreement with the in vitro data. The MCF MKM accurately describes the α and the RBE_10%_ trends for ^20^Ne ions. The α and RBE_10%_ values calculated by the MCF MKM for ^28^Si ions appear to underestimate the in vitro data. The LEM IV appears to systematically underestimate α and RBE_10%_ for both ^20^Ne and ^28^Si ions. Once the dispersion in the in vitro *ß* values for ^12^C and ^20^Ne ions is considered, we conclude that both models describe this quantity reasonably well. On the other hand, though the *ß* values calculated by the MCF MKM and LEM IV for ^28^Si ions are somehow similar, both models significantly overestimate the experimental trend of *ß*.

**Figure 8:**
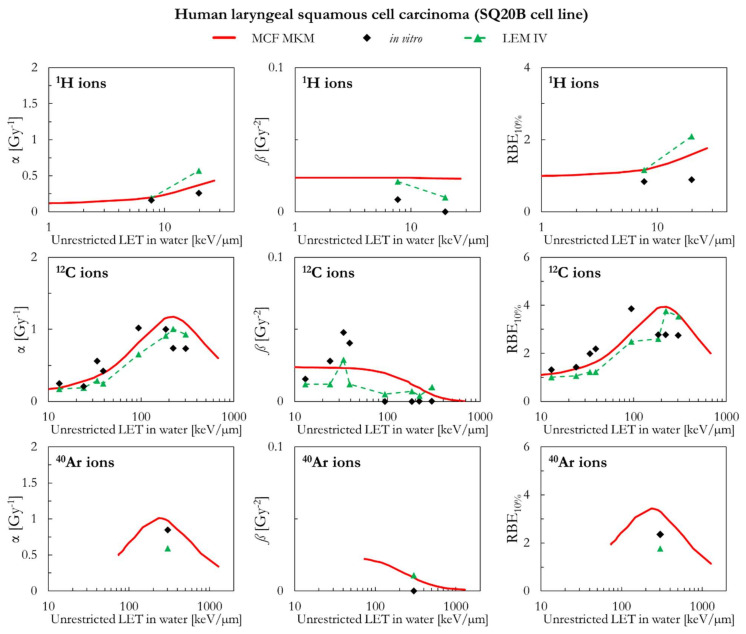
α, *ß*, and RBE_10%_ for the SQ20B cell line in case of ^1^H, ^12^C, and ^40^Ar ions: MCF MKM predictions compared with published in vitro data from PIDE 3.2 [34] and published LEM IV results [28].

The in silico and in vitro data for human laryngeal squamous cell carcinoma (SQ20B cell line) are compared in Figure 8. In case of ^1^H ions, the MCF MKM and the LEM IV seem to overestimate the in vitro α and RBE_10%_ values. The overestimation is larger for the LEM IV. Except for the two highest-LET in vitro entries for ^12^C ions, the MCF MKM seems to well reproduce the in vitro α values. On the other hand, the LEM IV results appear to be smaller. However, the LEM IV seems to better describe the *ß* trend as a function of the LET for ^12^C ions. The in vitro RBE_10%_ of ^12^C with LET < 100 keV/µm appears to be underestimated by both models, with the LEM IV showing the smaller RBE_10%_ values. The MCF MKM seems to reproduce the in vitro α entry for ^40^Ar ions reasonably well. Though larger than the corresponding in vitro entry, both models predict a similar *ß* value for ^40^Ar ions. The MCF MKM and the LEM IV, respectively, overestimates and underestimates the RBE_10%_ for ^40^Ar ions.

**Figure 9:**
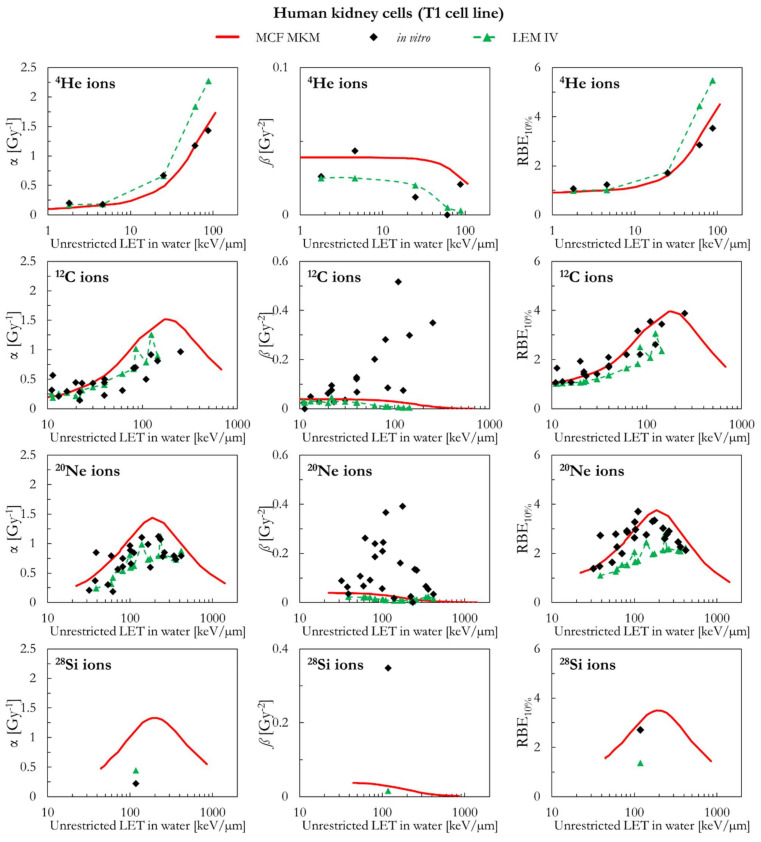
α, *ß*, and RBE_10%_ for the T1 cell line in case of ^4^He, ^12^C, ^20^Ne and ^28^Si ions: MCF MKM predictions compared with published in vitro data from PIDE 3.2 [34] and published LEM IV results [28].

**Figure 10:**
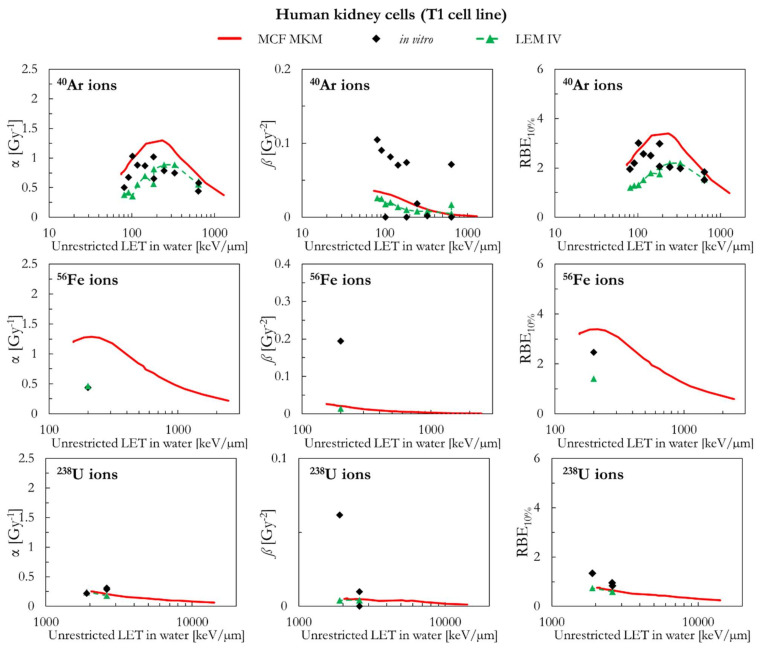
α, *ß*, and RBE_10%_ for the T1 cell line in case of ^40^Ar, ^56^Fe, and ^238^U ions: MCF MKM predictions compared with published in vitro data from PIDE 3.2 [34] and published LEM IV results [28].

The results of human kidney cells (T1 cell line) are plotted in Figure 9 (^4^He, ^12^C, ^20^Ne, and ^28^Si ions) and Figure 10 (^40^Ar, ^56^Fe, and ^238^U ions). In case of ^4^He ions, the MCF MKM seems to accurately reproduce the in vitro data. By contrast, the LEM IV appears to overestimate the α and RBE_10%_ in vitro data for ^4^He ions. For ions from ^12^C to ^56^Fe, the in vitro data are characterized by large values of *ß* (i.e., significantly higher than *ß*_ref_) which are underestimated by both models. Therefore, due to the previously discussed anti-correlation between α and *ß* [34,45], it would be expected that the models overestimate the in vitro α data. Indeed, this is the case for the MCF MKM that predicts α values larger than the in vitro ones. Nonetheless, the RBE_10%_ values computed with the MCF MKM are in satisfactory agreement with the in vitro results for these ions (^12^C to ^56^Fe). Interestingly, the α values calculated by the LEM IV for ions from ^12^C to ^56^Fe agree reasonably well with the in vitro data. However, the RBE_10%_ predicted by the LEM IV are systematically smaller than the in vitro data (^12^C to ^56^Fe ions). In case of ^238^U ions, the results of both models are in reasonable agreement with the in vitro data.

**Figure 11:**
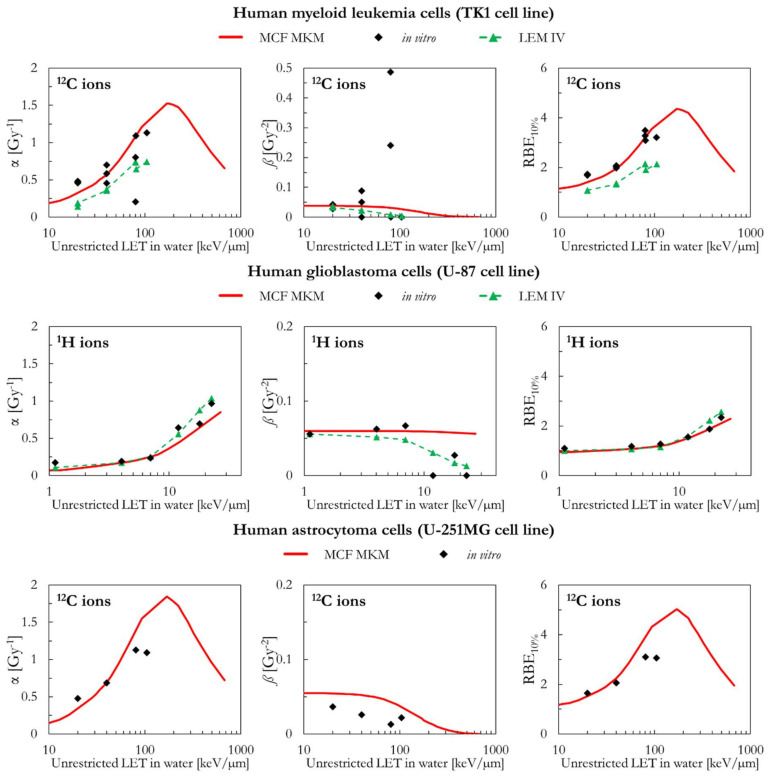
α, *ß*, and RBE_10%_ for the TK1, the U87, and the U251MG cell lines in case of ^1^H (U-87 cell line) and ^12^C (TK1 and U-251MG cell lines) ions: MCF MKM predictions compared with published in vitro data from PIDE 3.2 [34] and published LEM IV results [28].

As can be seen in Figure 11, the MCF MKM predictions for the carbon-irradiated human myeloid leukemia cells (TK1 cell line) are in good agreement with the in vitro data. In contrast, the α and RBE_10%_ results of the LEM IV are systematically lower than the corresponding in vitro ones. The spread in the in vitro *ß* data prevents from drawing any conclusion on which model better describes this quantity for the TK1 cell line. 

Figure 11 also includes the results of human glioblastoma cells (U-87 cell line) exposed to protons. Though the LET dependence of the LQM parameters seems to be best described by the LEM IV, the RBE_10%_ values calculated by both models are similar and in good agreement with the in vitro data. 

Finally, the MCF MKM predictions for human astrocytoma cells (U-251MG cell line) are compared to the carbon-exposed in vitro data in Figure 11. This cell line is characterized by an unusually low α_ref_ (=0.031 Gy^−1^), an unusually low α_ref_/*ß*_ref_ ratio (=0.56 Gy), and relatively large in vitro values of α for the ^12^C ions exposures (>0.4 Gy^−1^). Therefore, the in vitro RBE for low dose exposures (RBE_α_ = α/α_ref_) shows very large values (up to 35, Figure S15 in the Supplementary Materials). Though some deviations between the in silico and the in vitro data are present especially for the entry at LET > 100 keV/µm, it is surprising that the MCF MKM was able to reasonably predict most of the results of this unusual data series.

## 3. Materials and Methods

### 3.1. Relative Biological Effectiveness

The linear-quadratic model (LQM, Equation (1) [17,18]) was used to describe all clonogenic survival curves included in this article.

(1) $$S=\exp\left(-\alpha D-\beta D^{2}\right)$$

*S* is the surviving fraction as a function of the absorbed dose *D*, α and *ß* are exposure- and cell- specific fitting parameters.

The RBE for the surviving fraction *S* (RBE_S_) was calculated using Equation (2) [35]

(2) $$RBE_{S}=\frac{\alpha +\sqrt{\alpha^{2}-4\;\beta \ln\left(S\right)}}{\alpha_{\mathrm{ref}}+\sqrt{\alpha_{\mathrm{ref}}^{2}-4\;\beta_{\mathrm{ref}}\ln\left(S\right)}}$$

where α, *ß*, α_ref_ and *ß*_ref_ are the linear and quadratic terms of the LQM for the radiation under investigation and the reference photon exposure respectively.

### 3.2. Clonogenic Survival Data

#### 3.2.1. Particle Irradiation Data Ensemble

All in vitro clonogenic survival data were extracted from the Particle Irradiation Data Ensemble (PIDE [29]) version 3.2 (updated in November 2019 [34]). The database contains 1118 ion exposure entries for more than 100 cell lines. The data were collected from 115 papers published between 1966 and 2015. Corresponding survival curves for the reference photon exposures are also listed when available. All in vitro entries are for cell lines irradiated in normoxic conditions. 

The following physical information are listed for each entry: particle type, energy, LET in water, beam type (monoenergetic or a spread out Bragg peak), cell line details (name, human or rodent, tumor or healthy), and the cell cycle phase. Two sets of linear-quadratic terms are included in PIDE: the result of the fit performed by the authors of the original publication and by the PIDE team. When possible, the second set of LQM terms was preferred due to the systematic fit procedure by the PIDE team [29,35]). Uncertainty intervals are not provided for the clonogenic survival data in PIDE. 

#### 3.2.2. Initial Filtering

For consistency with the in silico calculations performed with monoenergetics ions, we discarded PIDE entries for cell exposures within spread out Bragg peaks. To avoid partial cell irradiations and the large uncertainties associated with very-low energy exposures, we discarded entries in case of ions with energy <1 MeV/n. Afterwards, since this work deals with asynchronized repair-competent cell lines only, we included only asynchronized PIDE entries for which at least one of the two LQM sets (original fit and PIDE fit) includes positive α_ref_ and *ß*_ref_ values. Similarly, we included PIDE entries for which at least one of the two LQM sets has a non-negative value of *ß* for the ion exposure. These negative *ß* values are supposed to be due to the presence of subpopulations of cells with different radiation resistance [34]. However, this is currently beyond the predictive capability of the MCF MKM and will likely be topic of future investigation. We also excluded 9 entries for which the only available set of LQM terms for the ion exposures listed α = 0 and a large value of *ß.* This initial filtering of PIDE led to a database containing 629 entries: 296 for human cell lines and 333 for rodent cell lines. 

The following paragraphs describe the process of in vitro data selection for both rodent and human cell lines. An overview on the cell lines included in this study (name of the cell line, type of cell, number of PIDE entries, ions used for the exposures) is given in Table 1. 

#### 3.2.3. Data Selection for Rodent Cell Lines

After the initial filtering, 333 PIDE entries were available for rodent cell lines. Since the results of Chinese hamster lung fibroblasts (V79 cell line) were topic of a previous investigation [33], the 211 PIDE entries for the V79 cell line were not included in this study. 

85 of the remaining 122 PIDE entries were in case of repair-competent Chinese hamster ovary cells (CHO and CHO-K1 cell lines), Chinese hamster peritoneal fibroblasts (B14FAF28 cell line), and mouse embryo fibroblasts (C3H10T1/2 cell line). All the B14FAF28 entries in PIDE were extracted from a single study [46] and are in case of irradiations with heavy ions (^40^Ar and heavier ions) with energy less than 10 MeV/n. However, in case of ions heavier than ^20^Ne, it was only possible to accurately compute the microdosimetric spectra for ions of energy equal or greater than 10 MeV/n (see Section 3.3.2). Therefore, the in vitro data for the B14FAF28 cell line were not included in the analysis. 

The remaining 37 rodent entries in PIDE were for 16 different cell lines. Cell lines with less than 3 entries for at least one ion were discarded. The remaining 14 PIDE entries were in case of rat intestinal epithelial fibroblasts (IEC-6 cell line), rat prostatic adenocarcinoma epithelial cells (R-3327-AT-1 cell line), epidermal cells of a newborn mice transformed with the carcinogenic dimethylbenzanthracene (PDV cell line), and squamous skin cancer cells obtained by injecting PDV cells in mice (PDV C57 cell line). Since the number of chromosomes and the radiosensitivity of the IEC-6 cell line were reported to significantly vary with the passage number [47], this cell line was not included in the analysis. Similarly, we excluded the PDV C57 cell line since we could not find karyotypic nor morphologic information. It was not possible to assume that the PDV C57 cells are similar to the ones of the parent PDV cell line because the former ones are significantly larger and with a more heterogenous morphology [48]. Since the R-3327-AT-1 cell line is named RAT-1 in PIDE and in the original publication [47], we use this abbreviation also in this article.

#### 3.2.4. Data Selection for Human Cell Lines

At first, PIDE entries for the human salivary gland tumor cells (HSG cell line), glioblastoma cells (A-172 cell line), and foreskin fibroblasts (AG01522 cell line) were excluded since part of other MCF MKM studies (HSG cell line in [33]; A-172 and AG01522 cell lines in [45]). Clonogenic survival data for 10 human cell lines were included in this study: the 8 cell lines with most entries in PIDE (listed below), cervical cancer cells (HeLa cell line), and astrocytoma cells (U-251MG cell line). The HeLa cell line was selected since it is the oldest immortalized human cell line, it is commonly used for cancer research, and because morphologic and karyotypic information are available in the literature. The U-251MG cell line was chosen due to its clinical relevance, the unusually low α_ref_/*ß*_ref_ ratio of ~0.6 Gy, and the availability of morphologic and karyotypic information in the literature. The 8 cell lines with most entries in PIDE were: kidney cells (T1 cell line), skin fibroblasts (NB1RGB cell line), laryngeal squamous cell carcinoma (SQ20B cell line), myeloid leukemia cells (TK1 cell line), leukemia cells (HL-60 cell line), fetal lung fibroblasts (HF19 cell line), mammary epithelial cells (M/10 cell line), and glioblastoma cells (U-87 cell line). Entries for U-87 MG cell line were not pooled together with the ones for the U-87 cell line because of their different radiosensitivity (i.e., α_ref_/*ß*_ref_ = 1.9 Gy for the U-87 cell line [49]; α_ref_/*ß*_ref_ = 6.8 Gy for the U-87 MG cell line [50]. This is likely due to the different origin of these two cell lines [39]. 

### 3.3. Biophysical Modeling

The MCF MKM [33] was used to predict the clonogenic survival curves. The linear and quadratic terms of the LQM (α and *ß*) are calculated using Equations (3) and (4) respectively.

(3) $$\alpha =\alpha_{0}\int \left(1+\frac{\beta_{0}}{\alpha_{0}}\;\frac{y}{\rho \;\pi \;r_{d}^{2}}\;\;\right)c\left(y\right)\;d\left(y\right)\;dy\;$$

(4) $$\beta =\beta_{0}{\left[\int c\left(y\right)d\left(y\right)\;dy\;\right]}^{2}$$

The correction factor *c(y)* accounts for the non-Poisson distribution of lethal lesions at high LET and is calculated with Equation (5).

(5) $$c\left(y\right)=\frac{1-\exp\left[-\alpha_{0}\left(1+\frac{\beta_{0}}{\alpha_{0}}\;\frac{y}{\rho \;\pi \;r_{d}^{2}}\;\right)\;\frac{y}{\rho \;\pi \;R_{n}^{2}}\;-\beta_{0}\;{\left(\frac{y}{\rho \;\pi \;R_{n}^{2}}\right)}^{2}\;\right]}{\alpha_{0}\left(1+\frac{\beta_{0}}{\alpha_{0}}\;\frac{y}{\rho \;\pi \;r_{d}^{2}}\;\right)\;\frac{y}{\rho \;\pi \;R_{n}^{2}}\;+\beta_{0}\;{\left(\frac{y}{\rho \;\pi \;R_{n}^{2}}\right)}^{2}}$$

*d(y)* is the dose probability density of the lineal energy *y*, α_0_ and *ß*_0_ are the LQM terms in the limit of *y* → *0*, $R_{n}$ is the mean radius of the cell nucleus, *r*_d_ is the mean radius of the subnuclear domains, and *ρ* is the density (=1 g/cm^3^). 

The equation used to assess α (Equation (3)) is an alternative implementation of the non-Poisson MKM [8]. While the non-Poisson MKM [8] is based on the calculation of the biological effect for the dose-mean lineal energy value of the microdosimetric spectrum, the MCF MKM calculates α as the dose-mean value of the biological effect over the microdosimetric spectrum [33]. In a somehow similar way as the clinical version of the LEM [51], the MCF MKM assumes that *ß* (LQM term describing the quadratic-dependence of the clonogenic survival with respect to dose) can be computed using a quadratic implementation (Equation (4)) of the correction factor *c(y)* (Equation (5)) used for the calculation of α [33].

#### 3.3.1. Model Parameters

In addition to the simulated microdosimetric spectra (Section 3.3.2), the MCF MKM requires these parameters as input for the RBE calculations: the mean radius of the spherical cell nucleus (*R*_n_), the mean radius of the spherical subnuclear domains (*r*_d_), the LQM terms for the reference photon exposure (α_ref_ and *ß*_ref_), and the LQM terms in the limit of *y* → *0* (α_0_ and *ß*_0_). No in vitro ion-exposure data were used at any point for the assessment of these parameters. All the model parameters are representative of population-mean characteristics [33] and are described below. The numerical values of *R*_n_, *r*_d_, α_ref_ and *ß*_ref_ used in the MCF MKM calculations are listed in Table 2.

##### Mean DNA Content of the Irradiated Population, Γ

At first, we introduce an additional parameter that is used for the assessment of *r*_d_ and, in some cases, also of *R*_n_: the mean DNA content of the irradiated population Γ. The latter quantity is calculated with Equation (6)

(6) Γ=γ p ξ 

where  γ is the normal DNA content for one set of chromosomes (3050 Mbp for humans, 2750 Mbp for rats, 2700 Mbp for Chinese hamsters, and 2650 Mbp for mice [14]), *p* is the ploidy number, and *ξ* is a factor accounting for the cell cycle distribution of the irradiated population (*ξ* = 4/3 for asynchronized cells [33]).

The ploidy number *p* is equal to 2 for healthy cell lines and for diploid cancer cells. Though mutated chromosomes might contain an abnormal amount of DNA, an approximated value of *p* for aneuploid cancer cells can be calculated with Equation (7)

(7) $$p=\frac{x}{x_{n}}$$

where *x* is the mean number of chromosomes in the aneuploid cell population and *x*_n_ is the number of chromosomes in a normal set (23 for humans, 21 for rats, 11 for Chinese hamsters, and 20 for mice [14]).

In this work, the ploidy number *p* was set to 2 for the healthy cell lines (C3H10T1/2, CHO and CHO-K1, M/10, NB1RGB, and T1) and for the myeloid leukemia cells (TK1 cell line) which were reported to be pseudodiploid [52]. For the aneuploid cancer cell lines the ploidy number was calculated with Equation (7). The number of chromosomes in the aneuploid cell population *x* was extracted from literature and it is equal to: 78 for the HeLa cell line [53], 44 for the HL-60 cell line [54], 62 for the PDV cell line [55], 59 for the RAT-1 cell line [47], 39.4 for the U-87 cell line [56], and 54.6 for the U-251MG cell line [56]. Since we could not find karyotypic information for the SQ20B cell line, we derived a value of 39.3 chromosomes as the mean of the number of chromosomes for similar head and neck squamous cell carcinoma cells [56].

##### Mean Radius of the Cell Nucleus, *R*_n_

Three approaches are used for the assessment of the mean radius of the cell nucleus *R*_n_. Details of the morphological information from literature can be found in Table S1 in the Supplementary Materials.

(1) Preferably, the radius of the cell nucleus is extracted from literature as determined by means of morphologic measurements for cells presenting a spherical nucleus (i.e., after trypsinization). This is the approach used in our previous work [33] dealing with Chinese hamster lung fibroblasts (V79 cell line) and human salivary glands tumor cells (HSG cell line). 

However, since this information is not always available, two additional strategies are used in this article. 

(2) The mean radius of the spherical nucleus (*R*_n_) can be assessed from the cross-sectional area of the nucleus measured in case of attached cells. Generally, *R*_n_ is calculated with Equation (8)

(8) $$R_{n}=\sqrt{\frac{A}{\pi }}$$

where *A* is the cross-sectional area of the nucleus. This approach implicitly assumes that the half-thickness of the nucleus is equal to the radius assessed with Equation (8). However, the nucleus of cells attached during the microscopic measurements is not spherical, but more similar to an oblate spheroid/ellipsoid. Published results indicate that the vertical semi-axis of the oblate spheroid/ellipsoid is roughly a third of the mean radius of the cross-sectional area of the nucleus [57,58,59,60]. Therefore, the volume of the spherical nucleus with radius equal to the one calculated with Equation (8) can be significantly larger than the one of the real (spheroid/ellipsoid-like) nucleus.

Thus, we introduce an alternative formula (Equation (9)) to calculate *R*_n_ as the radius of the sphere having the same volume of a spheroid with the equatorial radius equal to the one calculated with Equation (8) and the minor semi-axis equal to a third of the equatorial radius

(9) $$R_{n}=\frac{\sqrt{\frac{A}{\pi }}\;}{\sqrt[{3}]{3}}\approx 0.693\;\sqrt{\frac{A}{\pi }}\;$$

where *A* is the cross-sectional area of the nucleus.

**Figure 12:**
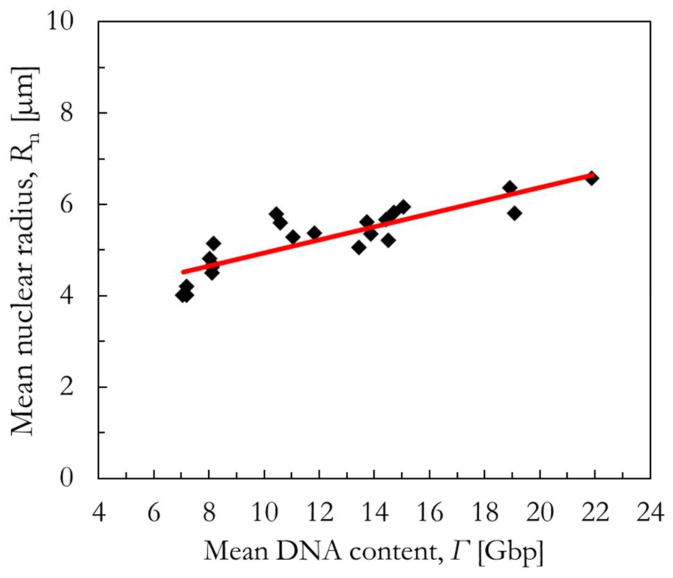
Phenomenological correlation (red line) between the mean nuclear radius and the mean DNA content of asynchronized cells (black diamonds). More details can be found in Table S1 in the Supplementary Materials.

(3) If also no cross-sectional area measurements are available, we propose a novel phenomenological correlation between the mean DNA content of an asynchronized population *Γ* and the mean radius of the cell nucleus *R*_n_ for asynchronized cells. In this regard, Figure 12 shows the mean radius of the cell nucleus *R*_n_ plotted as a function of the mean DNA content of 22 different asynchronized cell lines (details on the published morphological data [9,57,58,61,62,63,64,65,66,67,68,69] can be found in Table S1 in the Supplementary Materials). A phenomenological correlation was established between the two quantities as described in Equation (10). It is discouraged to extrapolate this correlation outside the investigated range of *Γ* [Gbp].

(10) $$R_{n}=3.5\;µm+0.144\;\frac{µm}{Gbp}\;\cdot \Gamma$$

##### Mean Radius of the Subnuclear Domains, *r*_n_

The subnuclear domains in MKMs are meant to represent the dimension of subnuclear structures where the accumulation of radiation-induce damage (likely DNA DSBs) is thought to correlate with cell death [12,26,70]. Previous studies suggest that these subnuclear structures are likely to be giant loops of chromatin containing approximately 2 Mbp of DNA [70,71,72]. 

Therefore, under the simplifying assumption that the DNA is homogenously distributed within the cell nucleus, we hypothesize that the MCF MKM subnuclear domains represent the average volume of the cell nucleus containing 2 Mbp of DNA. Thus, the mean radius of the subnuclear domains (*r*_d_) can be assessed with Equation (11) [33]:

(11) $$r_{d}=R_{n}\sqrt[{3}]{\frac{\lambda }{\Gamma }}$$

where $R_{n}\;$ is the average nuclear radius, λ is the amount of DNA in a chromatin substructure (2 Mbp), and Γ is the average DNA content of the irradiated cell population.

##### LQM Terms for the Reference Photon Exposure, *α*_ref_ and *ß*_ref_

If all PIDE entries for a specific cell line share the same photon reference irradiation, then α_ref_ and *ß*_ref_ are simply the α and *ß* values for that reference photon survival curve. Alternatively, as done in previous studies [31,33], in silico calculations representative of the average cell line radiosensitivity were performed. This was done to efficiently tackle the dispersion of a cell-specific in vitro dataset presenting entries extracted from an heterogenous pool of different publications. The average values of α_ref_ and *ß*_ref_ were calculated with Equations (12) and (13):

(12) $$\alpha_{ref}=\frac{\sum \alpha_{ref,\;i}\;n_{i}}{\sum n_{i}}$$

(13) $$\beta_{ref}=\frac{\sum \beta_{ref,\;i}\;n_{i}}{\sum n_{i}}$$

where α_ref,i_ and *ß*_ref,i_ are the LQM terms for a reference photon exposure, and *n*_i_ is the number of ion-exposure entries for that set of α_ref,i_ and *ß*_ref,i_. This weighted sum was used to prevent photon experiments with few ion-exposure entries from biasing the calculation of the average α_ref_ and *ß*_ref_. This approach was used for half of the cell lines included in this article, namely: CHO and CHO-K1, C3H10T1/2, HF19, NB1RGB, SQ20B, T1, and TK1.

##### LQM Terms in the Limit of *y* → 0, α_0_ and *ß*_0_

α_0_ and *ß*_0_ are determined by analyzing only photon in vitro data. Since the integral in Equation (5) is equal to ~1 for the reference photons, then *ß*_0_ = *ß*_ref_. 

Similarly, α_0_ can be calculated using Equation (14) [33]:

(14) $$\alpha_{0}=\alpha_{ref}-\;\beta_{0}\;\frac{{\bar{y}}_{D,ref}^{\;}}{\rho \;\pi \;r_{d}^{2}}$$

where ${\bar{y}}_{D,ref}^{\;}$ is the dose-mean lineal energy for the reference photon exposure. 

The dose-mean lineal energy ${\bar{y}}_{D}^{\;}$ is calculated as in Equation (15).

(15) $${\bar{y}}_{D}^{\;}=\;\int y\;d\left(y\right)\;dy$$

Since it is unpractical nor feasible to perform experiment-specific calculations of ${\bar{y}}_{D,ref}^{\;}$, representative simulated ${\bar{y}}_{D,ref}^{\;}$ values were used: 2.3 keV/µm for γ-rays (^60^Co and ^137^Cs) and energetic X-rays (6 MV), 4.3 keV/µm for less energetic X-rays (i.e., 200 kV X-rays) [33].

#### 3.3.2. Radiation Transport Simulations

The computation of the microdosimetric spectra and the LET follows the methodology of our previous study with the MCF MKM [33] and only a summary is given here. The lineal energy distributions were assessed with the microdosimetric function [73,74] implemented in the Particle and Heavy Ion Transport code System [75] version 3.2.4. The function was used to compute microdosimetric distributions in homogeneous conditions for spherical targets randomly placed around the particle track over an infinitesimal layer of water. Monoenergetic beams of ^1^H, ^4^He, ^7^Li, ^12^C, ^16^O, ^20^Ne, ^28^Si, ^40^Ar, ^56^Fe, ^238^U ions were simulated. The energy of the ions was equal to 1, 2, 3, 4, 5, 6, 7, 8, 9, 10, 20, 30, 40, 50, 60, 70, 80, 90, 100, 200, 300, 400, 500, 600, 700, 800, 900, 1000 MeV/n. The lineal energy spectra for ions heavier than ^20^Ne with energy lower 10 MeV/n were disregarded because of the presence of an inappropriate peak (personal communication with Tatsuhiko Sato, PHITS team leader). A logarithmic binning from 10^−2^ to 10^7^ keV/µm with 50 bins per decade was used to score the lineal energy spectra. The minimum energy deposition considered in the microdosimetric calculations was that for one event of one ionization only (10.9 eV [73]). 

In order to facilitate the comparison between the in silico and in vitro data, the unrestricted LET in water was assessed using the ATIMA model (http://web-docs.gsi.de/∼weick/atima/ (accessed on 5 September 2022) implemented in PHITS for the beams used to assess the microdosimetric spectra. Since the monoenergetic simulations were performed over an infinitesimal target, the simulated track- and dose- mean LET values were equivalent. By contrast, the in vitro experimental exposures are performed with quasi-monoenergetic beams. Consequently, track- and dose- mean LET values could differ. The inclusion of secondary fragments in the mean LET calculation and the use of different methods (stopping power tables, different LET models) significantly influence the LET results. Since details on the LET calculations were not always included in the publications from which the in vitro data were extracted and because different articles used different methodologies to calculate and average the LET, all results of this study are plotted as a function of a generic “unrestricted LET in water”.

## 4. Conclusions

The systematic benchmark performed in this work (10 ions from ^1^H to ^238^U, 14 cell lines with an α_ref_/*ß*_ref_ ratio ranging from ~0.6 to 30 Gy) suggests that the MCF MKM can accurately predict the in vitro clonogenic survival without requiring ion-irradiated in vitro data as input. This was possible thanks to several new strategies introduced to a priori determine the MCF MKM parameters by means of morphologic and karyotypic information.

Considering the differences between the modeling approaches (i.e., microdosimetry for the MCF MKM and amorphus track structure for the LEM IV) and the use of average radiosensitivity predictions for the MCF MKM (all LEM IV calculations are experiment specific), a reasonable agreement between the results of the models was found in most cases.

Future work with the MCF MKM is planned to include in the model dose-rate and fractionation effects, enhanced cell radioresistance due to hypoxia, and the transition of the survival curve to a constant slope at high doses. Additionally, we will investigate the possibility to extend the MCF MKM predictions to in vivo and patient data.

## Figures and Tables

**Table 1:** Details on the in vitro clonogenic survival data included in this article.

Cell Line Abbreviation	Species	Type of Cells	Number of Entries	Ions
C3H10T1/2	mouse	embryonic fibroblasts	20	^1^H, ^2^H, ^3^He, ^4^He, ^12^C, ^16^O, ^20^Ne, ^28^Si, ^40^Ar, ^56^Fe, ^238^U
CHO, CHO-K1	Chinese hamster	ovary epithelial cells	38	^12^C, ^16^O, ^20^Ne, ^40^Ar, ^56^Fe
HeLa	human	cervical cancer cells	4	^1^H, ^4^He
HF19	human	fetal lung fibroblasts	6	^1^H, ^12^C
HL-60	human	leukemia cells	7	^12^C, ^28^Si, ^56^Fe
M/10	human	mammary epithelial cells	6	^12^C
NB1RGB	human	skin fibroblasts	29	^12^C, ^20^Ne, ^28^Si
PDV	mouse	transformed epidermal cells	4	^1^H, ^7^Li
RAT-1	rat	prostatic adenocarcinoma epithelial cells	3	^12^C
SQ20B	human	laryngeal squamous cell carcinoma	11	^1^H, ^12^C, ^40^Ar
T1	human	kidney cells	63	^4^He, ^12^C, ^20^Ne, ^28^Si, ^40^Ar, ^56^Fe, ^238^U
TK1	human	myeloid leukemia cells	10	^12^C
U-87	human	glioblastoma cells	6	^1^H
U-251MG	human	astrocytoma cells	4	^12^C

**Table 2:** Cell-specific parameters used for the MCF MKM predictions. * = mean values calculated with Equations (12) and (13) for α_ref_ and *ß*_ref_ respectively. ** = calculated with the phenomenological correlation between the mean DNA content and the mean radius of the cell nucleus (Equation (10)). The cell-specific mean radius of the subnuclear domains was calculated with Equation (11).

Cell Line Abbreviation	*α* for the Reference Photon Exposure, $\alpha_{ref}$ [Gy^−1^]	*β* for the Reference Photon Exposure, $\beta_{ref}$ [Gy^−2^]	$\alpha_{ref}/\beta_{ref}$ [Gy]	Mean Radius of the Cell Nucleus, *R*_n_ [µm]	Mean Radius of the Subnuclear Domains, *r*_d_ [µm]
C3H10T1/2	0.173 *	0.389 *	4.44	4.0	0.26
CHO, CHO-K1	0.226 *	0.0231 *	9.78	4.2	0.27
HeLa	0.536	0.0278	19.3	5.6	0.29
HF19	0.557 *	0.0189 *	29.5	4.7 **	0.29
HL-60	0.315	0.0558	5.64	4.6 **	0.29
M/10	0.3	0.068	4.41	4.7 **	0.29
NB1RGB	0.476 *	0.0458 *	10.4	5.1	0.32
PDV	0.13	0.037	3.51	5.1 **	0.29
RAT-1	0.201	0.0266	7.53	5.0 **	0.29
SQ20B	0.122 *	0.0238 *	5.12	4.5 **	0.30
T1	0.159 *	0.0391 *	4.06	4.7 **	0.29
TK1	0.107 *	0.0384 *	2.79	4.7 **	0.29
U-87	0.106	0.0557	1.91	4.5 **	0.30
U-251MG	0.031	0.0551	0.563	4.9 **	0.29

## Data Availability

All MCF MKM results in this study are plotted of this article and in the Supplementary Materials. The in vitro data were extracted from the PIDE database [29,34]. The LEM IV data were extracted from the Supplementary Materials of [28].

## Supplementary Materials

The following supporting information can be downloaded at: https://www.mdpi.com/article/10.3390/ijms232012491/s1.

## Abbreviations

α	linear term of the linear-quadratic model of clonogenic survival
α_ref_	α for the reference photon exposure
*ß*	quadratic term of the linear-quadratic model of clonogenic survival
*ß*_ref_	*ß* for the reference photon exposure
DNA	deoxyribonucleic acid
DSB	double strand break
LEM	local effect model [6]
LEM IV	fourth version of the local effect model [26]
LET	linear energy transfer
LQM	linear-quadratic model of clonogenic survival [17,18]
MBM	mixed beam model [7]
MCF	Mayo Clinic Florida, Jacksonville, Florida, United States of America
MCF MKM	Mayo Clinic Florida microdosimetric kinetic model [33]
MKM	microdosimetric kinetic model [5]
modified MKM	modified microdosimetric kinetic model [9]
non-Poisson MKM	corrected version of the original microdosimetric kinetic model to account for the non-Poisson distribution of lethal lesions [8]
PHITS	Particle and Heavy Ion Transport code System [75]
PIDE	Particle Irradiation Data Ensemble [29,34]
RBE	relative biological effectiveness
RBE_S_	in vitro clonogenic cell survival RBE for the surviving fraction S
*r*_d_	mean radius of the subnuclear domains
*R*_n_	mean radius of the cell nucleus
