# Supplementary material for: The Mayo Clinic Florida Microdosimetric Kinetic Model of Clonogenic Survival: Application to Various Repair-Competent Rodent and Human Cell Lines
Source: Int J Mol Sci. 2022 Oct 18;23(20):12491. doi: 10.3390/ijms232012491 (PMC9604502; doi:10.3390/ijms232012491)
Supplement: Supplementary file 1 [file ijms-23-12491-s001.zip › ijms-1930280-supplementary.pdf]

SUPPLEMENTARY MATERIALS

to

The Mayo Clinic Florida microdosimetric kinetic model of clonogenic survival: application to various repair-competent rodent and human cell lines

Alessio Parisi [*], Chris J. Beltran, Keith M. Furutani

Department of Radiation Oncology, Mayo Clinic, Jacksonville, Florida, United States of America

[*] corresponding author, parisi.alessio@mayo.edu

SM1. Morphology of the cell nucleus

The mean radius of the spherical cell nucleus used in the MCF MKM calculations (R_n) was derived from the morphologic information of **Table SM1**.

R = radius of the spherical cell nucleus as reported in literature ($R_n = R$).

D = diameter of the spherical cell nucleus as reported in literature ($R_n = D/2$).

A = cross section area of the cell nucleus for fixed cells ($R_n = \frac{\sqrt{\frac{A}{\pi}}}{\sqrt[3]{3}}$).

V = volume of the cell nucleus ($R_n = \sqrt[3]{\frac{3}{4\,\pi}V}$).

Table S1. Morphology of the cell nucleus as reported in literature.

Cell line abbreviation	Reported quantity	Reference	Radius of the cell nucleus, R_n [μm]	Mean value of R_n, [μm]
Cell lines included in this article				
C3H10T1/2	D = 6-10 μm	Lammerdig, 2011	3.0-5.0	4.0
CHO, CHO-K1	A = 108 μm²	Weyrather et al., 1999	4.06	4.16
	A = 127 μm²	Konishi et al., 2005	4.41	
	V = 265 μm³	Gacsi et al., 2005	3.98	
HeLa	V = 690 μm³	Monier et al., 2000	5.48	5.63
	A = 219 μm²	Konishi et al., 2005	5.79	
NB1RGB	A = 172.3 μm²	Suzuki et al., 2000	5.13	
Additional cell lines used for the phenomenological correlation between mean DNA content and R_n (Equation 8 of the article)				
A-172	A = 209.1 μm²	Suzuki et al., 2000	5.66	
A-549	A = 182.3 μm²	Suzuki et al., 2000	5.28	
AG01522	A = 140 μm²	Azzam et al., 1998	4.63	4.57
	A = 133 μm²	Chaudhary et al., 2016	4.51	
Becker	A = 203.9 μm²	Suzuki et al., 2000	5.58	
C32TG	A = 166.6 μm²	Suzuki et al., 2000	5.05	
HFL-III	A = 142.9 μm²	Suzuki et al., 2000	4.68	
HSG	R(G1) = 4.1 μm	Kase et al., 2006	4.47*	
	R(G2) = 5.2 μm	Kase et al., 2006		
KNS-60	A = 263.8 μm²	Suzuki et al., 2000	6.35	
KNS-89	A = 187.3 μm²	Suzuki et al., 2000	5.35	
KS-1	A = 221.4 μm²	Suzuki et al., 2000	5.82	
LC-1 sq	A = 230.3 μm²	Suzuki et al., 2000	5.93	
Marcus	A = 187.9 μm²	Suzuki et al., 2000	5.36	
ONS-76	A = 176.9 μm²	Suzuki et al., 2000	5.20	
SF126	A = 150.9 μm²	Suzuki et al., 2000	4.80	
SK-MG-1	A = 218.1 μm²	Suzuki et al., 2000	5.78	
T98G	A = 282.5 μm²	Suzuki et al., 2000	6.57	
U-251MG(KO)	A = 219.9 μm²	Suzuki et al., 2000	5.80	
V79	R = 4.0 μm	Kassis et al., 1989	4.0	3.96
	D = 8.0 μm	Howell et al., 1991	4.0	
	A = 87.8 μm²	Weyrather et al., 1999	3.67	
	A = 113 μm²	Tracy et al., 2005	4.12	

* = the average R_n for the HSG cell line was calculated assuming that asynchronized cell lines can be approximated by a cell population with a 2:1 distribution of cells in G1 and G2 phase (see Parisi et al., 2022).

SM2. RBE for surviving fractions other than 10%

For better clarity, the comparison between *in vitro* data and *in silico* calculations (MCF MKM and published LEM IV results) was limited to α, *ß* and $RBE_{10\%}$ in **Figures 2-12** of the article. Nonetheless, as shown in a previous work with the MCF MKM (Parisi et al., 2022), the model can compute RBE values for other surviving fractions. For each cell line, **Figures SM1-14** shows examples of RBE values calculated with the MCF MKM for surviving fractions of 50% and 1%. Corresponding *in vitro* data from the Particle Irradiation Data Ensemble (PIDE, Friedrich et al., 2013) are included for comparison.

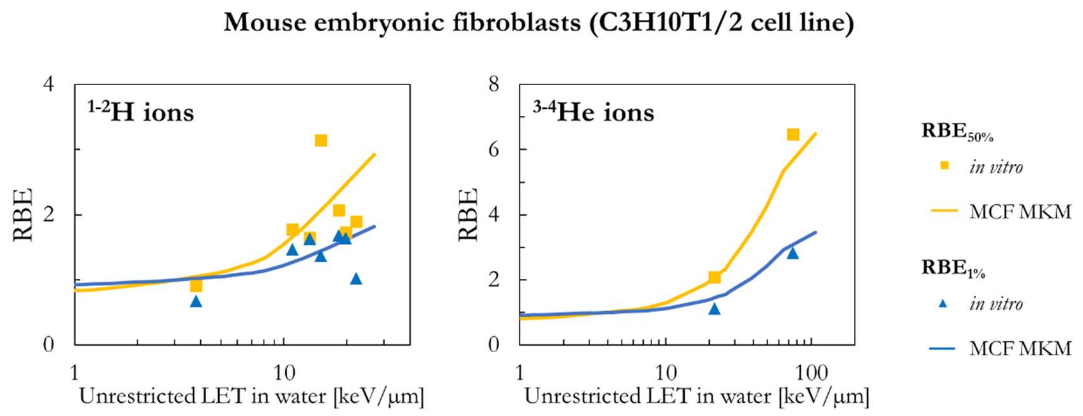

Figure S1. $RBE_{50\%}$ and $RBE_{1\%}$ for the C3H10T1/2 cell line: MCF MKM predictions compared with published *in vitro* data from PIDE 3.2 (Friedrich et al., 2021).

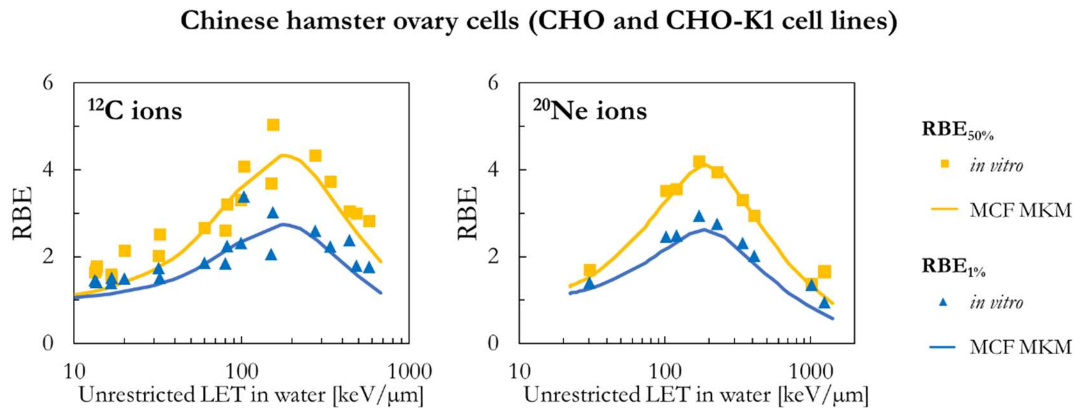

Figure S2. $RBE_{50\%}$ and $RBE_{1\%}$ for the CHO and CHO-K1 cell lines: MCF MKM predictions compared with published *in vitro* data from PIDE 3.2 (Friedrich et al., 2021).

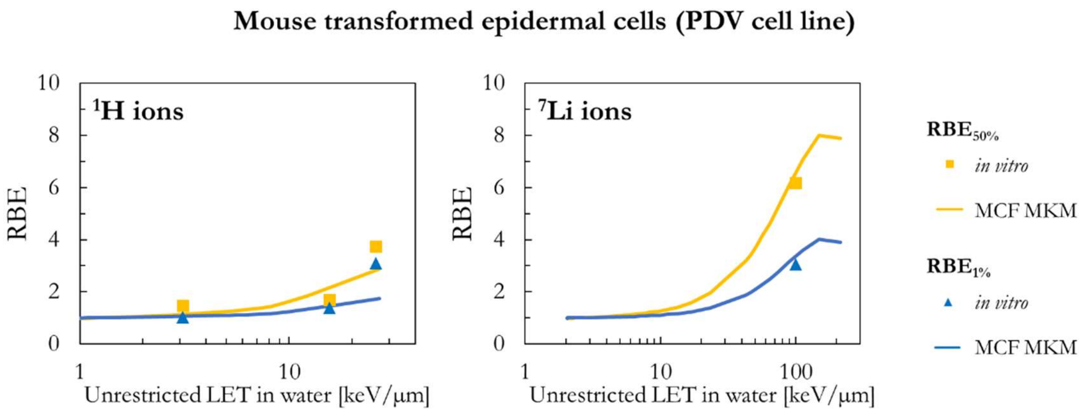

Figure S3. $RBE_{50\%}$ and $RBE_{1\%}$ for the PDV cell line: MCF MKM predictions compared with published *in vitro* data from PIDE 3.2 (Friedrich et al., 2021).

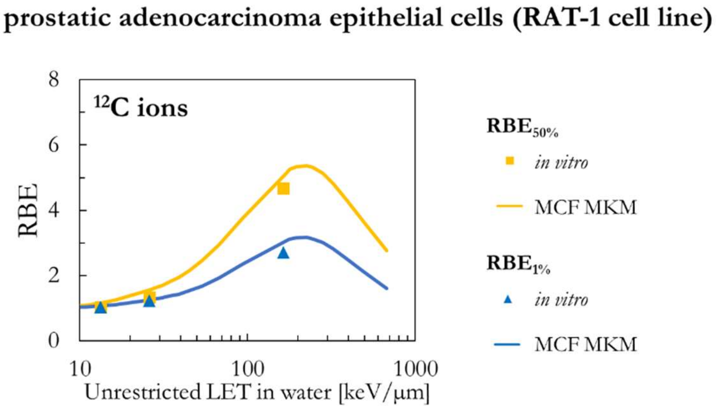

Figure S4. $RBE_{50\%}$ and $RBE_{1\%}$ for the RAT-1 cell line: MCF MKM predictions compared with published *in vitro* data from PIDE 3.2 (Friedrich et al., 2021).

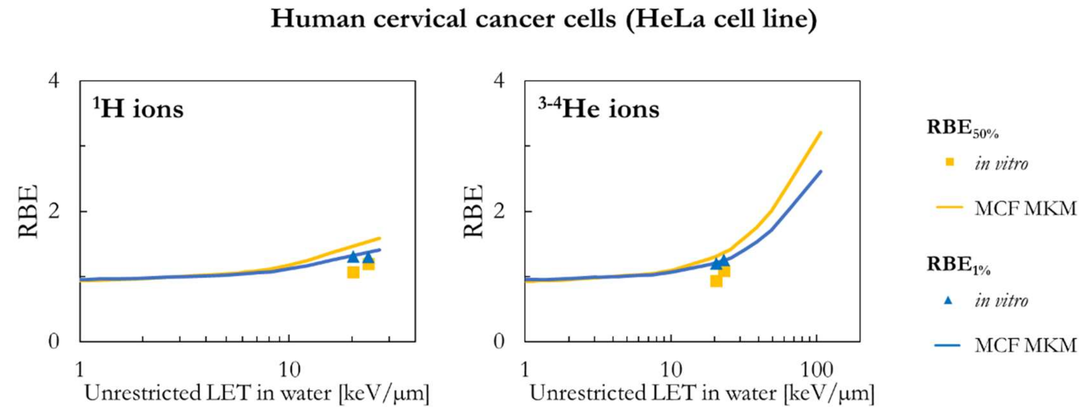

Figure S5. $RBE_{50\%}$ and $RBE_{1\%}$ for the HeLa cell line: MCF MKM predictions compared with published *in vitro* data from PIDE 3.2 (Friedrich et al., 2021).

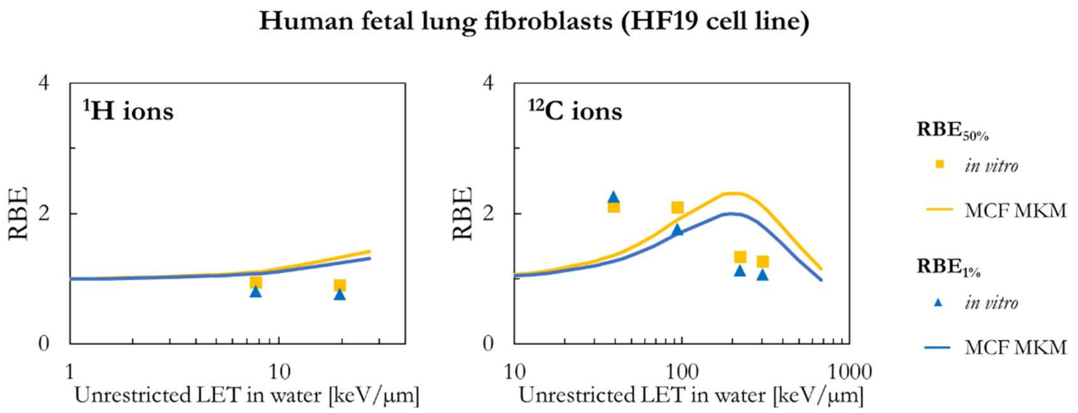

Figure S6. $RBE_{50\%}$ and $RBE_{1\%}$ for the HF-19 cell line: MCF MKM predictions compared with published *in vitro* data from PIDE 3.2 (Friedrich et al., 2021).

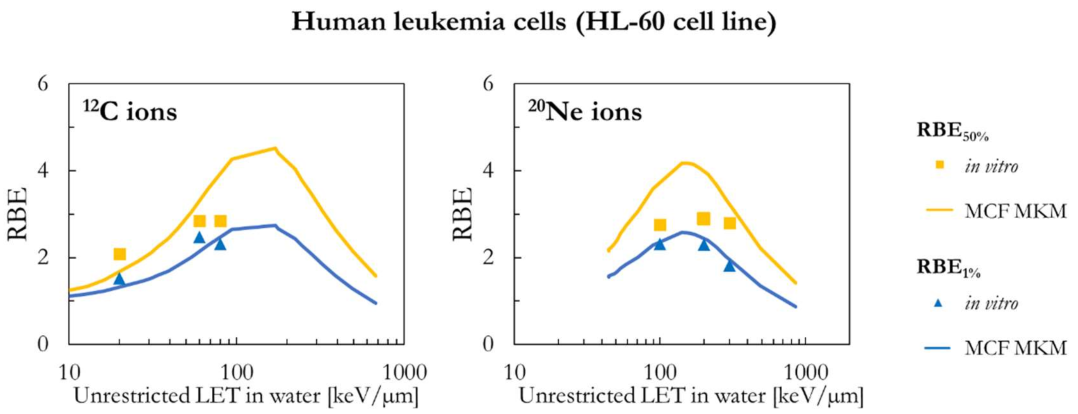

Figure S7. $RBE_{50\%}$ and $RBE_{1\%}$ for the HeLa cell line: MCF MKM predictions compared with published *in vitro* data from PIDE 3.2 (Friedrich et al., 2021).

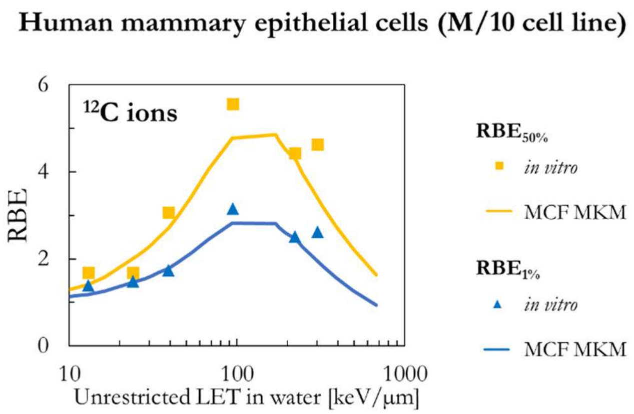

Figure S8. $RBE_{50\%}$ and $RBE_{1\%}$ for the M/10 cell line: MCF MKM predictions compared with published *in vitro* data from PIDE 3.2 (Friedrich et al., 2021).

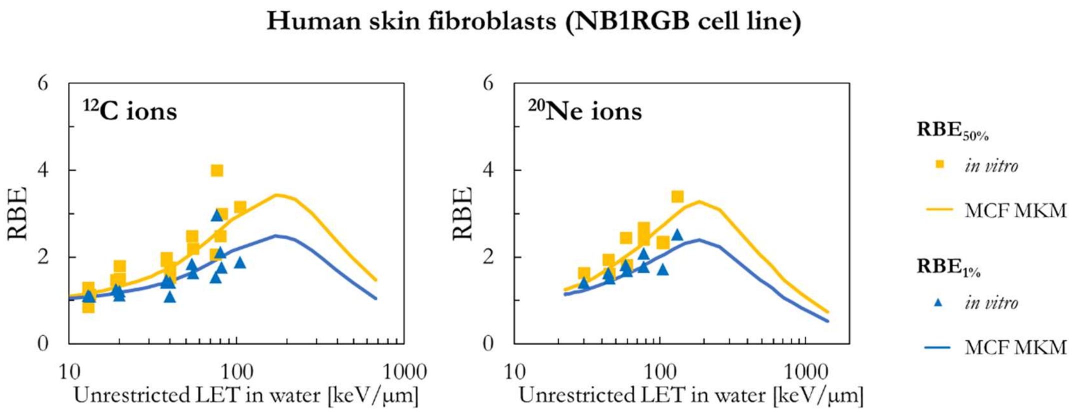

Figure S9. $RBE_{50\%}$ and $RBE_{1\%}$ for the NB1RGB cell line: MCF MKM predictions compared with published *in vitro* data from PIDE 3.2 (Friedrich et al., 2021).

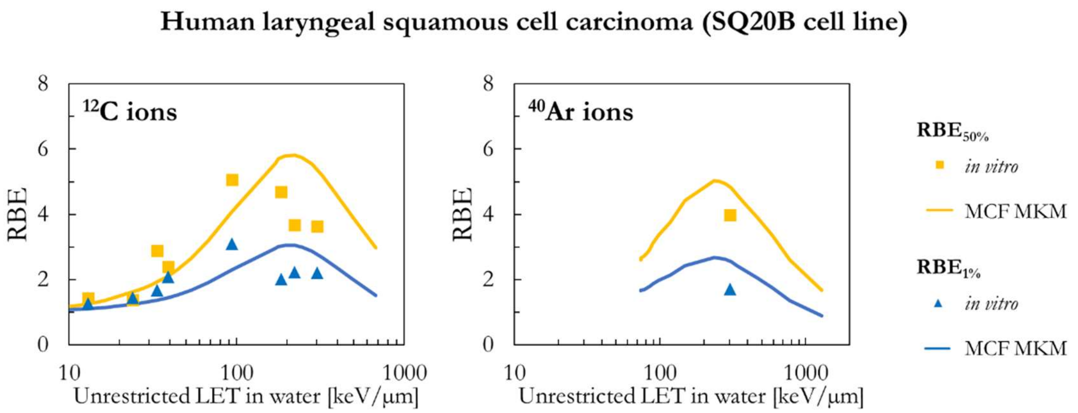

Figure S10. $RBE_{50\%}$ and $RBE_{1\%}$ for the SQ20B cell line: MCF MKM predictions compared with published *in vitro* data from PIDE 3.2 (Friedrich et al., 2021).

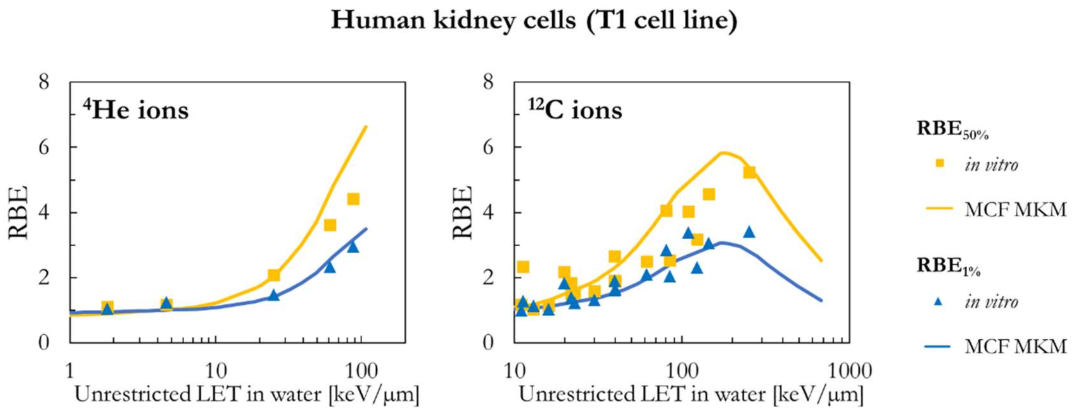

Figure S11. $RBE_{50\%}$ and $RBE_{1\%}$ for the T1 cell line: MCF MKM predictions compared with published *in vitro* data from PIDE 3.2 (Friedrich et al., 2021).

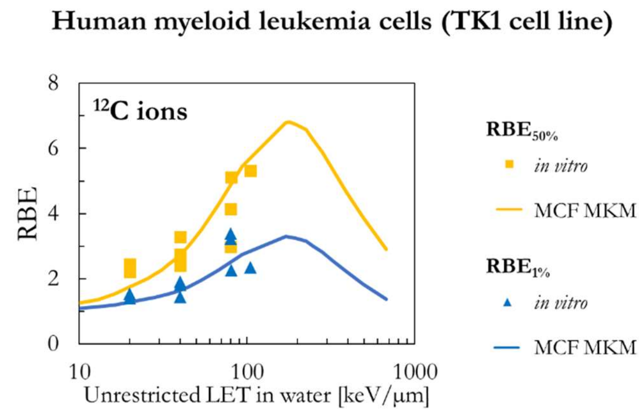

Figure S12. $RBE_{50\%}$ and $RBE_{1\%}$ for the TK1 cell line: MCF MKM predictions compared with published *in vitro* data from PIDE 3.2 (Friedrich et al., 2021).

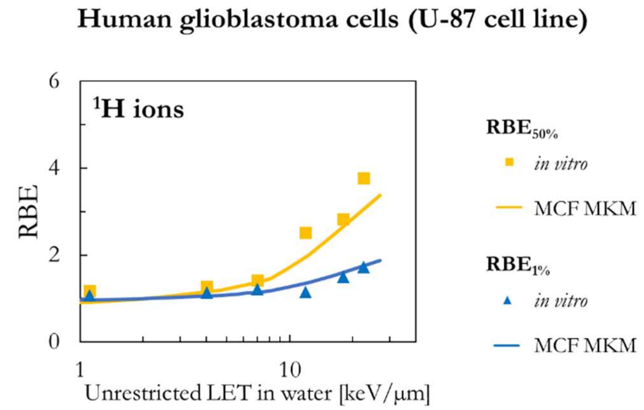

Figure S13. $RBE_{50\%}$ and $RBE_{1\%}$ for the U-87 cell line: MCF MKM predictions compared with published *in vitro* data from PIDE 3.2 (Friedrich et al., 2021).

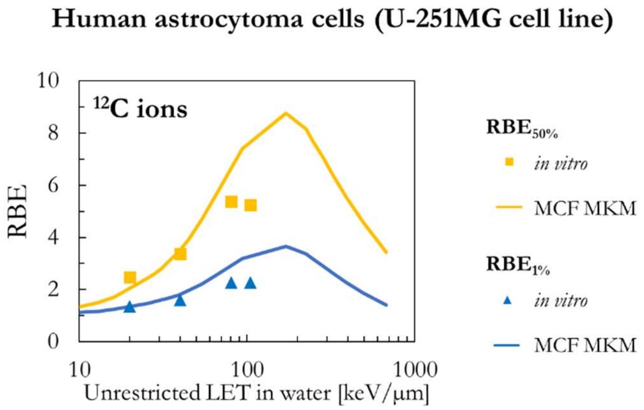

Figure S14. $RBE_{50\%}$ and $RBE_{1\%}$ for the U-251MG cell line: MCF MKM predictions compared with published *in vitro* data from PIDE 3.2 (Friedrich et al., 2021).

SM3. RBE_α for the U-251MG cell line

As discussed at the end of the **Discussion** section of the article, the *in vitro* data from PIDE (Friedrich et al., 2013) for the human astrocytoma cells (U-251MG cell line) are characterized by a relatively low value of α_{ref}, but relatively large values of α after exposures to ^{12}C ions. Therefore, large values of RBE_α ($RBE_\alpha = \alpha/\alpha_{ref}$) were obtained, as shown in **Figure SM15**.

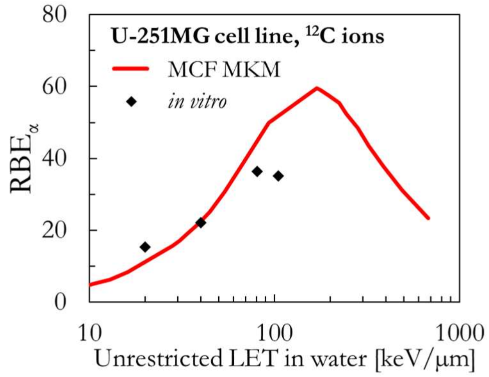

Figure S15. RBE_α for the U-251MG cell line: comparison between MKM MCF predictions and *in vitro* data.

References

- Azzam, E.I., De Toledo, S.M., Gooding, T. and Little, J.B., 1998. Intercellular communication is involved in the bystander regulation of gene expression in human cells exposed to very low fluences of alpha particles. Radiation research, 150(5), pp.497-504.
- Chaudhary, P., Marshall, T.I., Currell, F.J., Kacperek, A., Schettino, G. and Prise, K.M., 2016. Variations in the processing of DNA double-strand breaks along 60-MeV therapeutic proton beams. International Journal of Radiation Oncology* Biology* Physics, 95(1), pp.86-94.
- Friedrich, T., Scholz, U., Elsässer, T., Durante, M. and Scholz, M., 2013. Systematic analysis of RBE and related quantities using a database of cell survival experiments with ion beam irradiation. Journal of radiation research, 54(3), pp.494-514.
- Gacsi, M., Nagy, G., Pinter, G., Basnakian, A.G. and Banfalvi, G., 2005. Condensation of interphase chromatin in nuclei of synchronized Chinese hamster ovary (CHO-K1) cells. DNA and cell biology, 24(1), pp.43-53.
- Howell, R.W., Rao, D.V., Hou, D.Y., Narra, V.R. and Sastry, K.S., 1991. The question of relative biological effectiveness and quality factor for Auger emitters incorporated into proliferating mammalian cells. Radiation research, 128(3), pp.282-292.
- Kase, Y., Kanai, T., Matsumoto, Y., Furusawa, Y., Okamoto, H., Asaba, T., Sakama, M. and Shinoda, H., 2006. Microdosimetric measurements and estimation of human cell survival for heavy-ion beams. Radiation research, 166(4), pp.629-638.
- Kassis, A.I., Fayad, F., Kinsey, B.M., Sastry, K.S. and Adelstein, S.J., 1989. Radiotoxicity of an 125I-labeled DNA intercalator in mammalian cells. Radiation research, 118(2), pp.283-294

- Konishi, T., Takeyasu, A., Yasuda, N., Natsume, T., Nakajima, H., Matsumoto, K., Asuka, T., Sato, Y., Furusawa, Y. and Hieda, K., 2005. Number of Fe ion traversals through a cell nucleus for mammalian cell inactivation near the Bragg peak. Journal of radiation research, 46(4), pp.415-424.
- Lammerding, J., 2011. Mechanics of the nucleus. Comprehensive physiology, 1(2), p.783.
- Monier, K., Armas, J.C.G., Etteldorf, S., Ghazal, P. and Sullivan, K.F., 2000. Annexation of the interchromosomal space during viral infection. Nature cell biology, 2(9), pp.661-665.
- Parisi, A., Beltran, C.J. and Furutani, K.M., 2022. The Mayo Clinic Florida microdosimetric kinetic model of clonogenic survival: formalism and first benchmark against in vitro and in silico data. Physics in Medicine & Biology, 67(18), p.185013.
- Suzuki, M., Kase, Y., Yamaguchi, H., Kanai, T. and Ando, K., 2000. Relative biological effectiveness for cell-killing effect on various human cell lines irradiated with heavy-ion medical accelerator in Chiba (HIMAC) carbon-ion beams. International Journal of Radiation Oncology* Biology* Physics, 48(1), pp.241-250.
- Tracy, B.L., Stevens, D.L., Goodhead, D.T. and Hill, M.A., 2015. Variation in RBE for survival of V79-4 cells as a function of alpha-particle (helium ion) energy. Radiation research, 184(1), pp.33-45.
- Weyrather, W.K., Ritter, S., Scholz, M. and Kraft, G., 1999. RBE for carbon track-segment irradiation in cell lines of differing repair capacity. International journal of radiation biology, 75(11), pp.1357-1364.
